# The Bivalent COVID-19 Booster Immunization after Three Doses of Inactivated Vaccine Augments the Neutralizing Antibody Response against Circulating Omicron Sublineages

**DOI:** 10.3390/jcm12010146

**Published:** 2022-12-24

**Authors:** Qiaren He, Shiyu Sun, Xi Chen, Zhenxiang Hu, Yan Zhang, Hua Peng, Yang-Xin Fu, Jiaming Yang, Long Chen

**Affiliations:** 1 The Outpatient Department, Shaoguan Hospital of Traditional Chinese Medicine, Shaoguan 512026, China; 2 Guangzhou Laboratory, Guangzhou 510005, China; 3 Key Laboratory of Infection and Immunity, Institute of Biophysics, Chinese Academy of Sciences, Beijing 100101, China; 4 Department of Research and Development, Livzon Bio Inc., Zhuhai 519045, China; 5 Medical and Clinical Center, Livzon Pharmaceutical Group Inc., Zhuhai 519045, China; 6 Department of Basic Medical Sciences, School of Medicine, Tsinghua University, Beijing 100084, China

**Keywords:** BV-01-B5, V-01D-351, bivalent, fourth dose

## Abstract

A fourth dose of a COVID-19 vaccine has been recommended by a number of authorities due to waning immunity over time and the emergence of immune-escaping variants. Here, we evaluated the safety and immunogenicity of the bivalent BV-01-B5 or V-01D-351 or the prototype V-01 for heterologous boosting in three-dose inactivated COVID-19 vaccine (ICV) recipients, in comparison with ICV homologous boosting. One pilot study (NCT05583357) included 20 participants randomized at 1:1, either receiving V-01D-351 or CoronaVac. The other one (NCT05585567) recruited 36 participants randomized at 2:1, either receiving BV-01-B5 or V-01, respectively. BV-01-B5, V-01D-351, and V-01 were safe and well-tolerated as heterologous booster shots after three doses of ICV, with adverse reactions predominantly being mild and moderate in severity, similar to the safety profile of ICV boosters. The bivalent V-01D-351 and BV-01-B5 and prototype V-01 booster demonstrated remarkable cross-reactive immunogenicity against the prototype and multiple emerging variants of concern (VOCs), with the geometric mean ratio (versus CoronaVac) in particular being 31.3 (500 vs. 16), 12.0 (192 vs. 16) and 8.5 (136 vs.16) against BA.4/5 14 days after the booster, respectively. Taken together, the modified bivalent-formulation V-01 boosters induced robust neutralizing responses against multiple Omicron sublineages, better than V-01 and remarkably superior to ICV booster, without compromising the safety and tolerability.

## 1. Introduction

The ongoing COVID-19 pandemic remains a public concern globally, with Omicron and its descendent lineages (e.g., BA.5, BQ.1) outcompeting previous variants of concern (VOCs). The deployment of vaccines dramatically reduced the disease burden of COVID-19 [1]. However, Omicron and its descendent lineages that exhibited substantial immune escape, in addition to waning immunity over time, have posed critical challenges to current vaccination strategies. Most of the currently approved COVID-19 vaccines were designed based on the prototype SARS-CoV-2 (hereafter referred to as first-generation vaccines) and exhibited significantly decreased effectiveness against Omicron variants after the primary series and a single booster based on the evidence in clinical trials and real-world studies [2,3,4,5]. In addition, neutralizing antibody (NAb) titers against SARS-CoV-2 BA.2.12.1, BA.4, and BA.5 waned over time, indicative of reduced booster-induced immune protection [6]. SARS-CoV-2 variant-adapted vaccines (hereafter referred to as second-generation vaccines) use the same production/formulation process as first-generation vaccines to achieve safety equivalence. On the basis of the similar reactogenicity profile, this is an important strategy to tackle the ongoing pandemic, in particular as a booster that can induce stronger immune responses against multiple emerging variants.

As the immunity induced by vaccination or infection wanes over time, the COVID-19 vaccine booster approach has been widely adopted. Bivalent COVID-19 vaccines include a component of the previous strain (either prototype or pre-Omicron variants) to provide broad protection against COVID-19 and a component of the Omicron variant to provide better protection against COVID-19 caused by the dominant Omicron variant, and many bivalent COVID-19 vaccines based on different platforms (mRNA, inactivated, recombinant, adenovirus-vectored) are being developed. The purpose of bivalent vaccines used as a booster approach is to (1) induce a robust immune response against vaccine-targeting variants, (2) broaden cross-neutralization against other variants, including future variants, and (3) extend the immune durability. To date, bivalent Prototype/Omicron BA.1 and/or Prototype/Omicron BA.4/5 mRNA vaccines have gained emergency authorization from several countries, including the USA, Australia, Singapore, and several European countries. A bivalent vaccine mRNA-1273.211 (a bivalent mRNA vaccine targeting prototype SARS-CoV-2 and Beta strain, Moderna, 50 μg) inoculated after the primary series of mRNA-1273 (a monovalent mRNA vaccine targeting prototype SARS-CoV-2, Moderna, 100 μg) induced more potent, durable, and broad antibody responses against multiple variants, including variants not contained in the vaccine [7]. The NAb titers after the mRNA-1273.214 booster were superior to that after the mRNA-1273 booster, with the geometric ratio (GMR) of mRNA-1273.211/mRNA-1273 at 28 days after the booster being 1.28, 1.33, 1.77, 2.17, and at 180 days after the booster being 1.68, 2.74, 1.23, 2.32 against Prototype, Beta, Delta, and Omicron, respectively. A second booster dose (fourth dose) of the bivalent vaccine mRNA-1273.214 (a bivalent mRNA vaccine targeting prototype SARS-CoV-2 and Omicron BA.1 strain, Moderna, 50 μg) not only induced higher binding antibody responses against Alpha, Beta, Gamma, and Delta strains but also elicited potent NAb responses against the dominant Omicron BA.4 and BA.5 (BA.4/5) variants [8]. Similarly, it also elicited NAb responses against Omicron that were superior to the mRNA-1273 booster (50 μg), with the NAb GMT against the omicron BA.1 variant being 2372.4 after receipt of the mRNA-1273.214 booster and 1473.5 after receipt of the mRNA-1273 booster.

The inactivated COVID-19 vaccine (ICV) has been widely administered globally and has shown high protective efficacy against severe or hospitalized COVID-19 cases [9,10], e.g., 97.9% against severe or fatal outcomes after three-dose ICV (CoronaVac, SinoVac, 3 μg) in Hong Kong, and 78.8% against COVID-19 related hospitalization after three-dose CoronaVac in Chile. However, the humoral immune response induced by ICV is weak or even absent against the Omicron variant. Even after a first or second booster dosing (fourth dose) of ICV (BBIBP-CorV, Sinopharm, 4 μg), the NAb response against the Omicron variant is poor (40 times lower than the titers against the prototype), and the peak NAb level after the second booster was not better than that after the first booster [11]. What is worse, based on real-world data, it demonstrated low efficacy of about 15.9% or 17.9% against Omicron infection after the primary series or booster immunization of ICV during an outbreak in Shanghai, China, respectively [12], and it demonstrated an efficacy of 4.5% against laboratory-confirmed COVID-19 after three-dose ICV in Chile [10]. Thus, it is important to explore an appropriate booster strategy after three doses of ICV that can induce a robust NAb response against the emerging Omicron variants [13]. Heterologous boosting with recombinant proteins or mRNA vaccines was demonstrated to elicit a more robust neutralizing response or vaccine effectiveness than homologous boosting after the primary series of ICV [14,15,16,17]. For example, heterologous immunization with ICV (3 μg CoronaVac by SinoVac, or 4 μg BBIBP-CorV by Sinopharm) followed by the primary mRNA-booster (50 μg mRNA-1273 by Moderna, or 30 μg BNT162b2 by Pfizer/BioNTech) showed an enhanced ability to neutralize Beta, Delta and Omicron, with median NAb GMTs being 80, 320 and 20, respectively (versus 5 after ICV booster). Moreover, a fourth dose has been deployed by many countries to restore or boost immunity for protection against emerging SARS-CoV-2 variant infections. Incidence rates of confirmed SARS-CoV-2 infection after the fourth BNT162b2 vaccine (a monovalent mRNA vaccine targeting prototype SARS-CoV-2, Pfizer/BioNTech, 30 μg) were half those observed for the three-dose group [18]. The fourth dose restored the immunity to the peak response as three doses without safety concerns [19]. The incidence rates of solicited adverse reactions within seven days after the fourth dose (second booster) were similar for mRNA-1273.214 and mRNA-1273, and the majority of adverse reactions were mild to moderate [8]. Thus, a fourth dose is crucial to improve the protection from symptomatic COVID-19 without compromising safety, particularly after three doses of ICV, which demonstrated poor neutralizing activity against Omicron.

The efficacy of a SARS-CoV-2 booster vaccine, V-01 (a monovalent recombinant fusion protein vaccine targeting prototype SARS-CoV-2, Livzon, 10 μg), was evaluated in participants receiving a two-dose primary series of inactivated vaccine. This recombinant interferon-armed fusion protein vaccine (V-01) demonstrated a relative protective efficacy of 47.8% against symptomatic COVID-19 in an Omicron-dominating surveillance period [20]. Recently, this vaccine was approved for emergency use as a heterologous booster in China. Previously, we evaluated a bivalent booster approach with V-01D-351 (targeting Beta and Delta) in participants immunized with two-dose ICV, revealing that the bivalent booster sufficiently mounted an NAb response against prototype SARS-CoV-2 and the Delta variant, as well as Omicron BA.1, with an excellent 90-day immune durability [17]. Herein, we further conducted a pilot study to evaluate the immunogenicity and safety of two modified bivalent vaccine boosters, including the V-01D-351 and BV-01-B5 vaccines (targeting prototype SARS-CoV-2 and Omicron), in participants receiving three doses of ICV (CoronaVac).

## 2. Materials and Methods

### 2.1. Study Design and Participants

Two randomized, open-label pilot studies (Clincaltrials.gov identifier No.: NCT05583357 and NCT05585567) were conducted at Shaoguan Hospital of Chinese Medicine. These trials were designed to evaluate the immunogenicity and safety of V-01D-351 or BV-01-B5 as a heterologous booster in participants receiving three doses of ICV (3 μg of CoronaVac by SinoVac, or 4 μg of BBIBP-CorV by Sinopharm). The third and fourth dose interval was about 7–9 months. Participants with a known history of SARS-CoV-2 infection, severe, uncontrolled chronic disease, or other conditions that, according to the investigator, might interfere with the assessment of safety and immunogenicity or pose additional risks to participants were excluded from the study.

The two trials were conducted under the Good Clinical Practice Guidelines and Declaration of Helsinki. The Institutional Review Board of Shaoguan Hospital of Chinese Medicine approved the two trials. Each participant’s written informed consent form was obtained before any study-related activity.

### 2.2. Randomization and Masking

Blinding and masking were not applicable to the two open-label studies. However, the laboratory staff were masked to group allocation.

**Figure 1:**
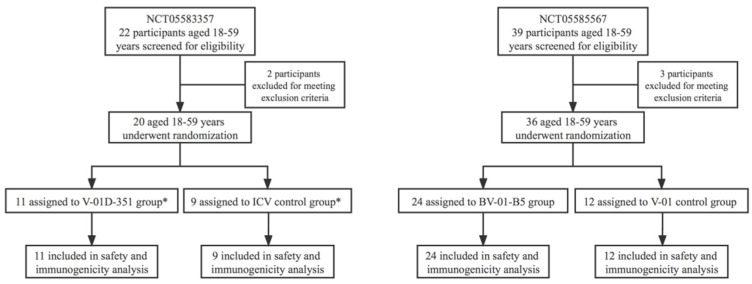
Trial flow diagram. * One participant allocated to the CoronaVac group received the V-01D-351.

As shown in Figure 1: in study NCT05583357, 20 participants were randomized at 1:1 either to receive V-01D-351 or CoronaVac; and in study NCT05585567, 36 participants were randomized at 2:1 either to receive BV-01-B5 or V-01. Randomization was performed by an independent statistician using SAS statistical software version 9.4 or above. Random numbers were allocated to eligible participants in the order of enrollment. Participants received the investigational vaccines according to the randomization table.

### 2.3. Procedures

The information on the investigational vaccines is summarized in Table 1. The design of the IFN-PADRE-RBD-Fc dimer was described in our previous studies. Briefly, the design of the variant-specific vaccine was based on the mutation sites on the RBD of emerging VOCs. In particular, the antigen of BV-01-B5 comprises 14 mutations from multiple VOCs, including Omicron and its descendent lineages. The mutation sites were finally selected for the vaccine design based on the probability of occurrence and the ability to perform immune escape, in combination with structural and computational analyses. The bivalent vaccine contained an equal amount of RBD from respective strains and was administered as a single booster dose through intramuscular injection.

Safety assessments: For both trials, participants were observed at the vaccination site for 30 min for immediate adverse events (AEs) following the booster vaccination. During the observation, participants were trained to complete the diary card to record any AEs experienced (including solicited local/systemic AEs and unsolicited AEs) within 7 days after the booster. On day 8, diary cards are collected and reviewed by the investigator, while contact cards were distributed to participants to record AEs (unsolicited AEs) within 8–28 days after the booster. Solicited local/systemic AEs, categorization, and grading of AEs were specified in our previous studies [21,22].

Immunogenicity assessments: Blood samples were drawn to determine NAb titers against prototype SARS-CoV-2 and circulating VOCs at baseline (pre-booster) and on days 7, 14, and 28 after the booster. The NAb titers against prototype SARS-CoV-2 and circulating VOCs were determined using VSV-based pseudovirus neutralizing assays (Supplementary Methods), which were conducted by LivzonBio, Inc. In the two trials, seroconversion was defined as the conversion of negative pre-boosting antibody levels to positive post-boosting levels, or at least a four-fold increase post-boosting levels relative to pre-boosting levels.

### 2.4. Study Objectives and Outcomes

In study NCT05583357, the primary objective was to evaluate the immunogenicity of the V-01D-351 booster compared to the ICV booster after three doses of ICV. The primary outcomes were the NAb titers against dominantly circulating VOCs at 14 and 28 days after the booster. The secondary objective was to evaluate the safety and immunogenicity of the V-01D-351 booster as compared to ICV: the secondary immunogenicity outcomes were the NAb titers against prototype SARS-CoV-2 and dominantly circulating VOCs at other specified time points after the booster; the secondary safety outcomes were immediate AEs within 30 min after the booster, solicited local and systemic AEs within 7 days, unsolicited AEs within 28 days after the booster vaccination, and possible severe adverse events.

In study NCT05585567, the primary objective was to evaluate the safety and immunogenicity of BV-01-B5 boosters compared to V-01. The secondary objective was to evaluate the immunogenicity against prototype SARS-CoV-2 and other VOCs after the BV-01-B5 and V-01 boosters. The outcomes were identical to those of study NCT05583357.

### 2.5. Statistical Analysis

The sample size of the two pilot studies was not determined based on a formal statistical hypothesis. Safety was evaluated in the safety set with all participants who received the booster dose. The immunogenicity analysis was performed in Per Protocol Set for Immunogenicity (I-PPS), including participants who had completed the booster immunization and had completed predefined blood samplings with available antibody results at pre-booster and 28 days after the booster with no major protocol deviations. The GMTs against prototype SARS-CoV-2 and VOCs after booster immunization with Clopper-Pearson 95% CIs were calculated. A covariance model was also used to normalize the NAb titers due to baseline differences. The model used log-transformed NAb titers at 7, 14, or 28 days after the booster as the dependent variable, the log-transformed baseline titers as the covariate, and the group as the fixed effect, and calculated the least square means of log-transformed NAb titers at 7, 14, or 28 days after the booster. Moreover, the geometric mean fold rises (GMFR) at each time point after the booster relative to pre-booster levels were statistically described. The frequencies and percentages of AEs, including overall AEs (solicited and unsolicited AEs), AEs related to vaccination (adverse reactions), AEs graded as grade 3 or worse, and AEs leading to participant withdrawal were presented. The χ^2^ test or Fisher’s exact test was used to analyze categorical safety data, and the *t*-test was used to compare log-transformed antibody titers between groups. Statistical analyses were performed with SAS 9.4 (SAS Institute Inc., San Diego, CA, USA and GraphPad Prism 8.0 (GraphPad Software Inc., San Diego, CA, USA).

## 3. Results

### 3.1. Trial Population

Between 15 August 2022 and 16 September 2022, a total of 22 participants were screened (two eligible participants were excluded after full recruitment was achieved), and 20 eligible participants were enrolled in study NCT05583357; a total of 39 participants were screened (one participant each was excluded due to a medical history of uncontrolled hypertension, tachycardia and planning to receive an HPV vaccine), and 36 eligible participants were enrolled in study NCT05585567 (Figure 1). The mean intervals (SD) between the first booster (third dose) and second booster (fourth dose) were 252.9 (7.3), 254.1 (7.3), 243.5 (24.8), and 242.4 (22.9) days, and the mean ages (SD) were 38.3 (8.6), 39.9 (7.8), 32.8 (10.2), 34.4 (10.4) years after V-01D-351, CoronaVac, BV-01-B5 and V-01 booster, respectively (Table 2). The baseline pseudovirus NAb titers (95% CI) were 168 (105–268), 97 (54–177), 34 (18–62), 33 (12–93) against prototype SARS-CoV-2, and 9 (7–11), 9 (7–12), 6 (5–7), 8 (3–20) against the dominantly circulating Omicron BA.4/5 variants in the V-01D-351, CoronaVac, BV-01-B5 and V-01 booster group, respectively (Table 2). The baseline characteristics were broadly similar across booster groups regarding age, booster intervals, and baseline NAb titers against the BA.4/5 variants. The baseline NAb titers against the prototype SARS-CoV-2 exhibited differences across groups. A covariance model was used to normalize the NAb titers after the booster in each group according to their baseline levels.

### 3.2. Safety

In the two trials, V-01D-351 and BV-01-B5 were safe and well-tolerated, with overall adverse reactions being mild and moderate in severity and mostly spontaneously recovered within 48 h after symptom onset. In study NCT05583357, a total of five (45.5%) and four (44.4%) participants reported adverse reactions after V-01D-351 and CoronaVac booster, respectively; in study NCT05585567, a total of twelve (50.0%) and three (25.0%) participants reported adverse reactions after BV-01-B5 and V-01 booster, respectively (Table S1). Generally, the adverse reactions were comparable across groups, resembling the safety profile of V-01D-351 or V-01 booster after a two-dose primary series of ICV. The most frequent solicited local adverse reactions were injection site pain, accounting for 27.3% (n = 3), 33.3% (n = 3), 45.8% (n = 11), and 16.7% (n = 2) of participants after V-01D-351, CoronaVac, BV-01-B5 and V-01 booster, respectively. Other solicited local adverse reactions, e.g., pruritus, swelling, redness, induration, and rash, were all anticipated for intramuscularly administered vaccines. The overall systemic adverse events were low, reported in ≤15% of participants in any group. There were no severe adverse events, adverse events of special interest, and adverse events leading to participant withdrawal in the two trials.

### 3.3. Immunogenicity

**Figure 2:**
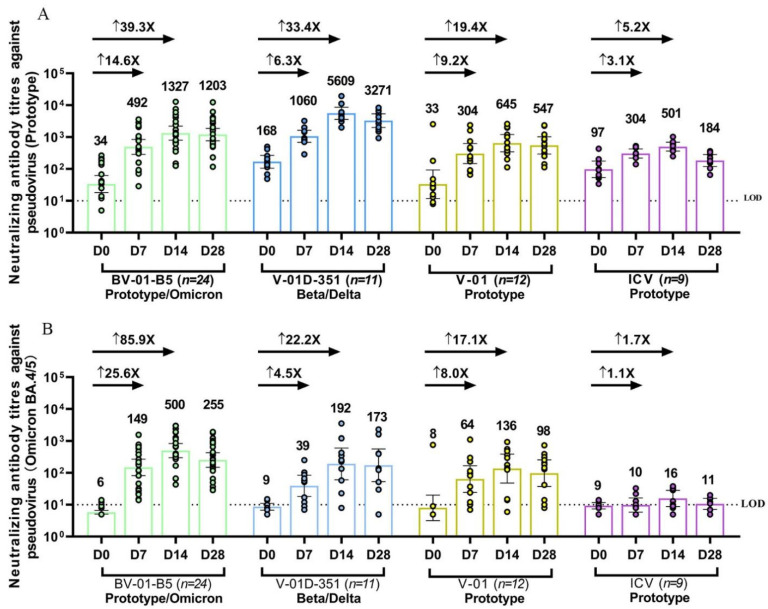
Pseudovirus-neutralizing antibody titers against prototype SARS-CoV-2 (**A**) and Omicron BA.4/5 strain (**B**) at baseline and 7, 14, 28 days after BV-01-B5, V-01D-351, V-01 or CoronaVac booster following three doses of inactivated vaccine. The individual data in each group are denoted by different colors, and bars denote geometric mean titer (95% CI). The lower dotted line denotes the limit of detection (LOD = 10). ↑ denotes the geometric mean fold rises (GMFR) of neutralizing antibody at 7, 14 days after booster from the baseline.

The fourth dose (second booster) of bivalent V-01D-351 or BV-01-B5 after three doses of ICV not only significantly restored the NAb responses against prototype SARS-CoV-2, but also elicited a much more robust cross-neutralizing activity against multiple circulating Omicron sublineages as compared to ICV vaccination (Figure 2). Since the blood samples from the two trials were detected head-to-head correspondingly, such as in the same test within one day for all samples on each time point (day 7, 14, and 28), we compared the immunogenicity of the bivalent vaccine booster approaches in two studies in one figure.

The NAb response against prototype SARS-CoV-2 before boosting (at 7–9 months after three doses of ICV) in most participants was still detectable. The fourth dose significantly elicited potent NAb responses with the peak levels at 14 days after the booster, remaining at high titers on D28 after the booster (Figure 2A). The antibody titers were 1327 (801–2198), 5609 (3617–8700), 645 (346–1202), 501 (363–692) at 14 days after the booster, and 1203 (769–1882), 3271 (1996–5361), 547 (293–1021), 184 (120–283) at 28 days after the booster in the BV-01-B5, V-01D-351, V-01, and CoronaVac booster group, respectively. The geometric mean fold rises (GMFRs) at 14 days after the booster from baseline were 39.3, 33.4, 19.4 in the BV-01-B5, V-01D-351, and V-01 booster groups relative to 5.2 in the CoronaVac booster group. It was observed that there were differences in the baseline NAb titers among the four groups, with higher titers in V-01D-351 and ICV than in BV-01-B5 and V-01. This baseline difference implied a potential impact on immune responses with subsequent booster vaccines, given that the individuals with a higher baseline titer could respond better to booster vaccines. As such, normalization using a covariance model was performed to obtain a more balanced data comparison. As shown in Table S2, the adjusted NAb GMTs against prototype SARS-CoV-2 were 1549, 3915, 756, 416 at 14 days after the booster, and 1380, 2373, 631, 156 at 28 days after the booster in the BV-01-B5, V-01D-351, V-01, and CoronaVac booster group, respectively. The GMR (versus CoronaVac) against prototype SARS-CoV-2 was 3.7, 9.4, 1.8 at 14 days after the booster, and 8.8, 15.2, 4.0 at 28 days after the booster in the BV-01-B5, V-01D-351, V-01 booster groups relative to the CoronaVac booster group, respectively. The vaccination-elicited NAb response showed a consistent trend before and after normalization in terms of the baseline NAb GMTs against prototype SARS-CoV-2.

Regarding the NAb response against BA.4/5, NAb GMTs were undetectable for almost all participants at 7–9 months after three doses of ICV. The fourth dose of V-01 and its bivalent-formulation vaccines elicited considerable NAb responses against a dominant Omicron BA.4/5 strain, even though the NAb activity against BA.4/5 was much lower than that of the prototype (Figure 2B). In contrast, boosting with CoronaVac had little effect on NAb activity against BA.4/5. A heterologous booster following three doses of ICV increased the NAb titers to 149 (82–270), 39 (18–84), and 64 (25–168) versus 10 (6–16) at 7 days, 500 (299–838), 192 (61–606), and 136 (48–387) versus 16 (9–28) at 14 days, and 255 (150–433), 173 (53–566), and 98 (37–258) versus 11 (7–16) at 28 days after the booster in the V-01D-351, BV-01-B5, and V-01 booster groups versus the CoronaVac group. It is noted that the Omicron-containing bivalent vaccine BV-01-B5 induced the highest NAb responses against the Omicron BA.4/5, followed by Beta-Delta specific V-01D-351 and prototype-based V-01, which were significantly higher than that in the CoronaVac group. The GMFRs 14 days after the booster from baseline were 85.9, 22.2, and 17.1 in the BV-01-B5, V-01D-351, and V-01 booster groups compared to 1.7 in the CoronaVac booster group. Similar to that of prototype SARS-CoV-2, NAb titers against BA.4/5 were also normalized in terms of baseline for data comparison. As shown in Table S2, there was a minor difference between normalized NAb GMTs and original GMTs, as the baseline NAb titers against Omicron BA.4/5 were comparable among all groups. Additionally, the vaccination-elicited NAb response to BA.4/5 showed a consistent trend before and after normalization.

**Figure 3:**
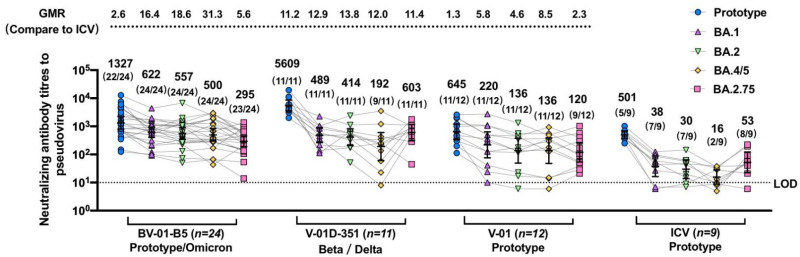
Pseudovirus neutralization titers against prototype SARS-CoV-2 and multiple Omicron sublineages for sera collected 14 days after the booster vaccination. The sera were from individuals vaccinated with the fourth dose of BV-01-B5, V-01D-351, V-01, or CoronaVac after three doses of inactivated vaccine. For all panels, the values above the symbols denote the geometric mean titer, the numbers in parentheses denote the seroconversion rates (a negative baseline titer (<5) to positive conversion (≥5), and at least four-fold increases from a positive baseline titer), and bars denote the geometric mean titer (95% CI). The numbers over the upper dotted line denote the geometric mean ratio (compared to CoronaVac) against multiple Omicron sublineages. The lower dotted line denotes the limit of detection (LOD = 10).

A heterologous booster dose of the bivalent vaccine V-01D-351 or BV-01-B5 after three doses of ICV demonstrated cross-reactive immunogenicity against prototype and multiple emerging VOCs (Figure 3). At 14 days after the booster, the geometric mean ratio (versus CoronaVac) was 2.6 (1327 vs. 501), 11.2 (5609 vs. 501), and 1.3 (645 vs. 501) against prototype SARS-CoV-2, 16.4 (622 vs. 38), 12.9 (489 vs. 38), and 5.8 (220 vs. 38) against Omicron BA.1, 18.6 (557 vs. 30), 13.8 (414 vs. 30), and 4.6 (136 vs. 30) against Omicron BA.2, 31.3 (500 vs. 16), 12.0 (192 vs. 16), and 8.5 (136 vs. 16) against Omicron BA.4/5, and 5.6 (295 vs. 53), 11.4 (603 vs. 53), and 2.3 (120 vs. 53) against Omicron BA.2.75 in the BV-01-B5, V-01D-351, V-01 booster groups relative to the CoronaVac booster group, respectively. A similar trend was also observed in the geometric mean ratio (versus CoronaVac) at 28 days after the booster (Figure S1). The highest NAb response against Omicron was observed in the BV-01-B5 booster group, followed by V-01D-351 and V-01, which was all superior to that in the CoronaVac booster group. Most participants in the bivalent V-01D-351 and BV-01-B5 groups showed positive seroconversion against the emerging Omicron sublineages, ranging from 81.8% to 100% (Figure 3 and Table S3) at 14 days after the booster. While the homogenous CoronaVac booster elicited little or weak NAb response against the Omicron variant, the heterologous V-01D-351, BV-01-B5, and V-01 booster broadened the neutralizing responses to the Omicron sublineages.

## 4. Discussion

The preliminary findings from the two trials indicate that the modified V-01 bivalent-formulation vaccines BV-01-B5 and V-01D-351 as fourth doses were safe and immunogenic in participants who received three doses of ICV 7–9 months earlier. The heterologous booster of variant-containing bivalent BV-01-B5 and V-01D-351 not only induced a superior immunity than the homologous CoronaVac booster against the prototype SARS-CoV-2 but also expanded the breadth of immunity to the emerging Omicron sublineages. Although the immunity-correlated protection has not been fully validated, abundant evidence showed that NAb titers correlate highly with protective efficacy against COVID-19 [23,24]. Therefore, the bivalent BV-01-B5 and V-01D-351 boosters have a high potential to provide good protective efficacy in the Omicron-dominated COVID-19 pandemic, which warrants further testing in clinical trials of a larger sample size. Additionally, even though V-01 does not contain variant-adapted components, the heterologous V-01 booster can achieve a much higher NAb titer against Omicron sublineages than that after homogenous ICV booster with poor neutralizing capability against Omicron. This result is consistent with the BNT162b2 as a primary or second booster based on the prototype, but also demonstrated effectiveness against SARS-CoV-2 Omicron BA.4/5 [25]. This implies that we could use the first-generation vaccines or second-generation V-01 vaccines as the fourth dose (the second booster). Two generations of V-01 vaccines may help to deal with the unforeseen and unpredictable situation of the ongoing pandemic and the national immunization strategy, as it will take time to obtain sufficient clinical data on humans for the second-generation vaccines.

Multiple bivalent Prototype/Omicron booster vaccines have gained provisional regulatory approval since August 2022, including bivalent Prototype/Omicron BA.1 and Prototype/Omicron BA.4/5 vaccines. Our results demonstrate that BV-01-B5 exhibited the best neutralizing capability against multiple Omicron sublineages, as well as a favorable neutralizing capability against the prototype SARS-CoV-2. This bivalent strategy in the design of new vaccines targets both current variants and prototypes to induce cross-reactivity against multiple variants. The antigen targeting Omicron in BV-01-B5 was developed using a chimeric protein design to present the optimal neutralizing epitopes of Omicron sublineages. The mutation sites on antigens were determined based on the probability of occurrence and the ability to perform immune escape, further guided by structural and computational analyses. A similar design was also used in a mutation-integrated trimeric vaccine, which showed a broad immune response against 23 SARS-CoV-2 variants [26]. Given the uncertainties of future evolution, our design strategy is successful in terms of the inclusion of the various immune-related Omicron mutations in the updated compositions of BV-01-B5 that achieved broader neutralizing activity against multiple circulating Omicron strains. BV-01-B5 contains an antigen component that best resembles neutralizing epitopes of Omicron, the most antigenically distinct VOCs, compared to the Delta/Beta bivalent vaccine V-01D-351. There has been plenty of evidence to indicate that a modified Omicron-containing vaccine composition would likely provide improved neutralizing capability or effectiveness against emerging VOCs. Previous studies indicated that BA.1 boosting by infection after two vaccine doses elicits greater breadth against Omicron variants than three doses of vaccines based on the prototype strain [27], and BA.1 or BA.2 infection after vaccination increases antibody titers against Omicron variants [28]. Additionally, a bivalent Omicron-containing booster has been shown to elicit superior neutralizing titers against Omicron as well as higher neutralizing titers against the prototype strain than the first-generation mRNA vaccine [8].

To the best of our knowledge, this is one of the first of a few studies to explore the safety and immunogenicity of heterologous boosters (fourth dose) after three doses of ICV. According to the findings in this study and in our previous study on heterologous boosters, it has been demonstrated that both V-01 and V-01D-351 boosters could induce remarkably higher NAb responses than an ICV homologous booster, no matter the prior vaccination procedure with two-dose or three-dose ICV [18]. Our findings indicated that maximal immunity was almost reached after a single heterologous booster irrespective of repeated ICV doses, inconsistent with another study [19]. Nevertheless, the maximal immunity to be achieved and the marginal benefits of using further heterologous boosters following the primary series of ICV are yet to be explored. Moreover, a study found that the peak RBD-NAb level induced by the fourth dose of ICV was inferior to the peak of the third dose of ICV, which increased the NAb titers against WT by 19 times and against Omicron by 2.9 times, and concluded that an updated vaccine with more diverse epitopes capable of inducing NAbs against VOCs should be a future direction for boosters [13]. The NAb response against the Omicron variant with an ICV homologous booster was much lower than that with a heterologous booster (85.9, 22.2, and 17.1 times for the BV-01-B5, V-01D-351, and V-01 booster, respectively) in our study for the currently dominant Omicron BA.4/5 in China. In terms of immune response over time, NAb responses against both prototype and Omicron BA.4/5 tended to decline at 28 days compared with that at 14 days after boosting. There were differences in the rate of titer decrease from day 14 to day 28 among the four groups. For prototype SAR-CoV-2, the NAb response to the ICV booster seemed to wane faster than the other heterologous boosters. The rapid decline observed in the CoronaVac booster group may be attributable to the increase of non-neutralizing responses to the NTD domain after the fourth dose accompanied by down-regulation of the neutralizing response to RBD [13]. With regard to Omicron BA.4/5, the NAb response to BV-01-B5 (bivalent vaccine targeting prototype and BA.5) might decline more rapidly than V-01 and V-01D-351 (bivalent vaccine targeting delta and beta). Our preliminary explanation for this decline (approximately 50%) of antibody titers against Omicron BA.4/5 in the bivalent vaccine booster group, particularly the BA.4/5 adapted BV-01-B5 booster, is given below and needs further verification. The recall of immune responses against conserved epitopes of prototype and VOCs may contribute to a larger proportion of NAb responses against BA.4/5 [29], while the de novo NAb responses directly against RBD epitopes against BA.4/5 may decline rapidly after a single booster, which may be restored by a second VOC-adapted bivalent vaccine booster.

The overall incidence rates of adverse reactions after the fourth dose following three doses of ICV were quite similar to those after the third dose following two doses of ICV. The incidence rates were comparable across heterologous booster groups with no additional side effects observed to those after the third dose and comparable to the homogenous CoronaVac booster. Additionally, the fourth dose did not seem to increase the intensity of adverse reactions, which were predominantly mild and moderate in severity. The adverse events were anticipated for intramuscularly inoculated vaccines using a traditional aluminum adjuvant. The most common adverse reactions were pain followed by redness, reported in over 15 percent of participants in any group. There were no additional safety concerns after the fourth dose, consistent with another study [18].

Our studies have several limitations. Firstly, the two studies have a relatively small sample size and were not fully blinded. Hence, the results should be interpreted with caution. Even though they are pilot studies, the preliminary immunogenicity data are valuable for the development of second-generation vaccines in terms of booster strategies to fight against the ongoing pandemic. A phaseII/III study for the bivalent vaccine with a larger sample size will be conducted, based on statistical hypotheses in regard to immunogenicity bridging (superiority against Omicron and inferiority against the prototype). Second, the durability of humoral immunogenicity has not been investigated in this study but will be updated in the follow-up study. Furthermore, not being able to include some currently circulating strains is another limitation of our study. We only detected the NAb titers of some strains circulating before or during our study period. These emerging circulating sublineages of VOCs, such as BA.4.6 and BQ.1, would be included in our future studies with a larger sample size [30,31]. Finally, the baseline NAb titers against prototype SARS-CoV-2 were not comparable across groups, which may be caused mainly by the small sample size, as well as different recruitment times leading to an insufficient balance of randomization. Thus, the results of the NAb titers after the booster should be appropriately interpreted. We have used a covariance model to minimize the immunological baseline differences and represented the adjusted data in our results. We will try to minimize the differences in the baseline immunity in our future studies by using designs with a larger sample size and appropriate methods of randomization.

## 5. Conclusions

The bivalent BV-01-B5 and V-01D-351 boosters after three doses of inactivated vaccine were safe and well-tolerated, with no additional safety concerns. The bivalent boosters induced robust NAb responses against multiple Omicron sublineages, better than V-01 and remarkably superior to the ICV booster. BV-01-B5 showed the best immunogenicity with regard to the BA.4/5 sub-lineage. Our findings provide valuable evidence for a bivalent strategy using computational and structural antigen design to elicit cross-neutralizing activity against emerging SARS-CoV-2 variants.

## Figures and Tables

**Table 1:** Information on the investigational vaccine.

Vaccine Name	Dose	Design	RBD Sequence
V-01	10 μg	IFN-PADRE-RBD-Fc dimer	RBD from prototype strain
V-01D-351	10 + 10 μg		RBD from Beta (K417N, E484K and N501Y) and Delta (L452R and T478K), 1:1 mixture
BV-01-B5	10 + 10 μg		RBD from prototype strain and Omicron (14 mutation sites on RBD selected by the probability of occurrence and the ability of immune escape, then undergo further structural and computational analyses), 1:1 mixture

**Table 2:** Baseline characteristics of participants.

	NCT05583357		NCT05585567	
	V-01D-351 (n = 11)	CoronaVac (n = 9)	BV-01-B5 (n = 24)	V-01 (n = 12)
Age (years)				
Mean (SD)	38.3 (8.6)	39.9 (7.8)	32.8 (10.2)	34.4 (10.4)
Median	40	40	30	35.5
Min, Max	27, 53	27, 53	20, 55	21, 55
Sex (%)				
Male	2 (18.2)	5 (55.6)	11 (45.8)	8 (66.7)
Female	9 (81.8)	4 (44.4)	13 (54.2)	4 (33.3)
Prime-boost interval (days)				
Mean (SD)	252.9 (7.3)	254.1 (7.3)	243.5 (24.8)	242.4 (22.9)
Median	255	256	242.5	246.5
Min, Max	243, 265	245, 265	160, 269	196, 268
Baseline neutralizing antibody GMTs (95% CI)				
Against prototype SARS-CoV-2	168 (105–268)	97 (54–177)	34 (18–62)	33 (12–93)
Against Omicron BA.4/5	9 (7–11)	9 (7–12)	6 (5–7)	8 (3–20)

## Data Availability

Not applicable.

## Supplementary Materials

The following supporting information can be downloaded at https://www.mdpi.com/article/10.3390/jcm12010146/s1, Table S1: Overall adverse reactions, solicited local and systemic adverse reactions following the V-01D-351, BV-01-B5, V-01 or CoronaVac booster; Table S2: Normalized Geometric mean titer (GMT) against prototype SARS-CoV-2 and Omicron BA.4/5 in V-01D-351, BV-01-B5, V-01 or CoronaVac booster group; Table S3: Seroconversion rate against prototype SARS-CoV-2 and multiple Omicron sublineages in the V-01D-351, BV-01-B5, V-01 or CoronaVac booster group; Figure S1: Pseudovirus neutralization titers against prototype SARS-CoV-2 and multiple Omicron sublineages for sera collected 28 days after the booster vaccination. The sera were from individuals vaccinated with a fourth dose of BV-01-B5, V-01D-351, V-01, or CoronaVac after a three-dose inactivated vaccine.
