# Supplementary material for: The Bivalent COVID-19 Booster Immunization after Three Doses of Inactivated Vaccine Augments the Neutralizing Antibody Response against Circulating Omicron Sublineages
Source: J Clin Med. 2022 Dec 24;12(1):146. doi: 10.3390/jcm12010146 (PMC9821285; doi:10.3390/jcm12010146)
Supplement: Supplementary file 1 [file jcm-12-00146-s001.zip › jcm-2039771-supplementary.pdf]

Journal of
Clinical Medicine

MDPI

Table S1. Overall adverse reactions, solicited local and systemic adverse reactions following V-01D-351, BV-01-B5, V-01 or CoronaVac booster.

Adverse reactions	NCT05583357		NCT05585567	
	**V-01D-351 (n=11)**	**CoronaVac (n=9)**	**BV-01-B5 (n=24)**	**V-01 (n=12)**
**Overall adverse reactions within 28 days after booster**				
Any	5 (45.5)	4 (44.4)	12 (50)	3 (25)
Vaccination-related	5 (45.5)	4 (44.4)	12 (50)	2 (16.7)
Grade 3 or worse	1 (9.1)	0	0	1 (8.3)
Vaccination related SAEs or AESIs within 0-28 days				
SAEs	0	0	0	0
AESIs	0	0	0	0
**Solicited local adverse reactions**				
Pain	3 (27.3)	3 (33.3)	11 (45.8)	2 (16.7)
Pruritus	2 (18.2)	0	0	0
Swelling	1 (9.1)	1 (11.1)	1 (4.2)	0
Redness	3 (27.3)	1 (11.1)	2 (8.3)	0
Induration	1 (9.1)	1 (11.1)	1 (4.2)	0
Rash	1 (9.1)	0	0	0
**Solicited systemic adverse reactions**				
Fever	0	1 (11.1)	1 (4.2)	1 (8.3)
Acute allergic reactions	1 (9.1)	0	0	0
Fatigue	0	1 (11.1)	3 (12.5)	1 (8.3)
Diarrhoea	0	0	1 (4.2)	0
Muscle pain	0	0	2 (8.3)	1 (8.3)
Headaches	0	0	3 (12.5)	1 (8.3)
Nausea	0	0	1 (4.2)	0
Cough	0	0	2 (8.3)	0
Anorexia	0	0	1 (4.2)	0

Data are presented as cases of adverse events or reactions (%).

Table S2. Normalized Geometric mean titer (GMT) against prototype SARS-CoV-2 and Omicron BA.4/5 in V-01D-351, BV-01-B5, V-01 or CoronaVac booster group.

	NCT05583357		NCT05585567	
**Normalized GMT**	**V-01D-351 (n=11)**	**CoronaVac (n=9)**	**BV-01-B5 (n=24)**	**V-01 (n=12)**
**Prototype**				
**D7**	649.2 (362.3, 1163.1)	236.5 (128.6, 434.8)	607.8 (416.4, 887.3)	378.2 (223.2, 640.9)
**D14**	3915.2 (2196.7, 6977.8)	416.5 (227.7, 761.6)	1548.9 (1064.7, 2253.4)	756.1 (448.3, 1275.3)

**D28**	2372.7 (1360.1, 4139.3)	156.2 (87.4, 279.4)	1380.5 (962.2, 1980.6)	630.9 (381.4, 1043.6)
		**Omicron BA.4/5**		
**D7**	35.2 (16.6, 74.4)	8.4 (3.7, 19.4)	170.7 (102, 285.6)	60.6 (29.7, 123.8)
**D14**	181.6 (79, 417.3)	14.5 (5.8, 36.6)	537.3 (303.4, 951.7)	132 (59.7, 291.8)
**D28**	164 (72.3, 372.1)	9.9 (4, 24.5)	273.2 (155.6, 479.6)	95.1 (43.6, 207.7)

Data are normalized using a covariance model and are presented as GMT (95% CI).

Table S3. Seroconversion rate against prototype SARS-CoV-2 and multiple Omicron subvariants in V-01D-351, BV-01-B5, V-01 or CoronaVac booster group.

	**NCT05583357**		**NCT05585567**	
**Seroconversion rate (%)**	**V-01D-351 (n=11)**	**CoronaVac (n=9)**	**BV-01-B5 (n=24)**	**V-01 (n=12)**
		**Day 14 after booster**		
**Prototype**	100 (71.5, 100)	55.6 (21.2, 86.3)	91.7 (73.0, 99.0)	91.7 (61.5, 99.8)
**BA.1**	100 (71.5, 100)	77.8 (40.0, 97.2)	100 (85.8, 100)	91.7 (61.5, 99.8)
**BA.2**	100 (71.5, 100)	77.8 (40.0, 97.2)	100 (85.8, 100)	91.7 (61.5, 99.8)
**BA.4/5**	81.8 (48.2, 97.7)	22.2 (2.8, 60.0)	100 (85.8, 100)	91.7 (61.5, 99.8)
**BA.2.75**	100 (71.5, 100)	88.9 (51.8, 99.7)	95.8 (78.9, 99.9)	75.0 (42.8, 94.5)
		**Day 28 after booster**		
**Prototype**	100 (71.5, 100)	22.2 (2.8, 60.0)	87.5 (67.6, 97.3)	91.7 (61.5, 99.8)
**BA.1**	90.9 (58.7, 99.8)	44.4 (13.7, 78.8)	100 (85.8, 100)	75.0 (42.8, 94.5)
**BA.2**	90.9 (58.7, 99.8)	33.3 (7.5, 70.1)	95.8 (78.9, 99.9)	83.3 (51.6, 97.9)
**BA.4/5**	72.7 (39.0, 94.0)	0	95.8 (78.9, 99.9)	91.7 (61.5, 99.8)
**BA.2.75**	90.9 (58.7, 99.8)	77.8 (40, 97.2)	95.8 (78.9, 99.9)	75.0 (42.8, 94.5)

Data are presented as seroconversion rate (95% CI).

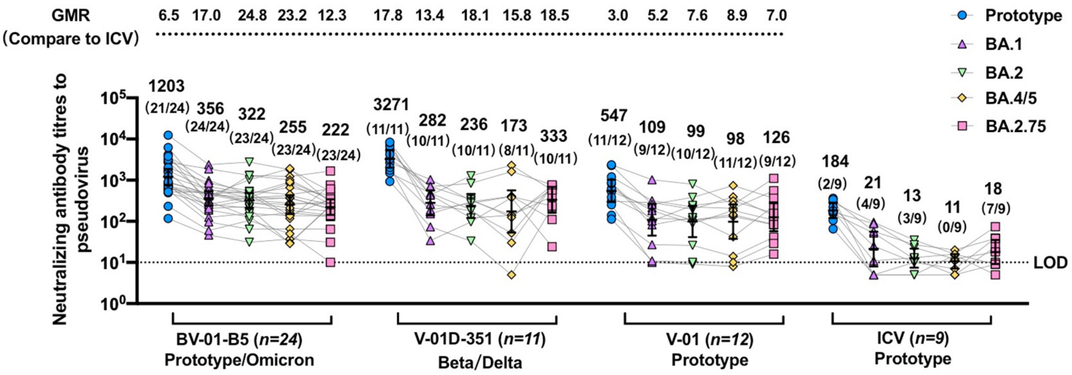

Figure S1. Pesudovirus neutralization titers against prototype SARS-CoV-2 and mul-tiple Omicron sub-lineages for sera collected 28 days after the booster vaccination. The sera were from individuals vaccinated with the fourth dose of BV-01-B5, V-01D-351, V-01, or CoronaVac after three-dose inactivated vaccine. *For all panels, values above the symbols denote geometric mean titer, the numbers in parentheses denote the seroconversion rates [a negative baseline titer (<5) to positive conversion (≥5), and at least four-fold increases from a positive baseline titer], and bars denote geometric mean titer (95%CI). The numbers over the upper dotted line denote the geometric mean ratio (compared to CoronaVac) against multiple Omicron subvariants. The lower dotted line denotes the limit of detection (LOD=10).*

Supplementary Methods

SARS-CoV-2 pseudovirus neutralization assay

Pseudovirus neutralization assay was performed using the VSV-based luciferase reporter SARS-CoV-2 pseudoviruses bearing prototype, omicron BA.1, BA.2, BA.4/5, and BA.2.75 subvariants spike protein, which were provided by Beijing Yunling Pharmaceutical Biotechnology Development Co. Ltd. The SARS-CoV-2 pseudotyped virus neutralization test began with 3-fold serially diluted heat-inactivated serum samples, which started at 1:5 and then mixed with a certain amount 650 $TCID_{50}$ of pseudotyped virus for about 1h. Then the mixture was added to 293-hACE2 cells (2×10^4 per well). After that, the target cells were incubated for 24 h, and the amount of pseudotyped virus entering the target cells was calculated by detecting the expression of luciferase. The 50% neutralization titer ($pVNT_{50}$) was calculated by GraphPadPrism 8.0 and was defined as the reciprocal of serum dilution at which the relative light units (RUL) were reduced by 50% compared with the virus control wells. Samples with values ≥ 5 were defined as seropositive.
